# On the ground in Western Africa: from the outbreak to the elapse of Ebola

**DOI:** 10.1007/s13238-016-0305-2

**Published:** 2016-08-09

**Authors:** William J. Liu

**Affiliations:** National Institute for Viral Disease Control and Prevention, Chinese Center for Disease Control and Prevention, Beijing, China

I was sitting in the chair of the airplane back to Beijing when I started writing the memoirs to recollect my three journeys to Western Africa. All of the trips were related to the Ebola epidemic and covered different stages, from the outbreak peak to the post-Ebola era. Each journey was taken for a different task, but all of them represented the urgent and specific support from the Chinese Center for Disease Control and Prevention (China CDC) during the Ebola epidemic in Western African.

On the August 8th, 2014, the World Health Organization (WHO) declared the Ebola outbreak in West Africa a Public Health Emergency of International Concern (PHEIC) (http://who.int/mediacentre/news/statements/2014/ebola-20140808/en/). Three days later, together with two colleagues, I was on the ground of Conakry (Fig. 1), the capital of Guinea, in which country the first Ebola case was traced back to the end of 2013 (Baize et al., 2014).

**Figure 1:**
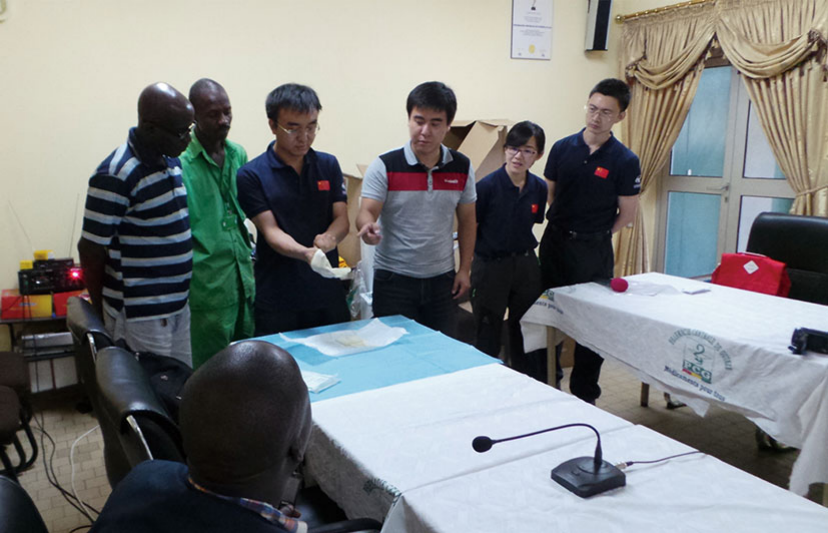
Training of the local health workers in Guinea to use the freely offered sanitary and biological protection supplies from China

As the first international support force during this epidemic, China rapidly reacted to help the three major affected countries, Guinea, Sierra Leone and Liberia, to fight against Ebola. Three experts were sent to each of these countries, respectively, to train the local health workers to use freely offered sanitary and biological protection supplies. The training went smoothly under the active cooperation of local health workers, and the supplies were soon delivered to the Ebola Treatment Units (ETUs) and the diagnostic laboratories in Guinea. This was the first time that China has sent public health specialists to countries outside of Asia to support disease control.

In September, 2014, as the Ebola-related support to Western Africa went deeper, additional Chinese specialists were sent to Sierra Leone. Clinical treatment teams in ETUs, Ebola test teams based in a mobile biological safety level 3 laboratory (BSL-3 or P3 lab) and public health training teams working in the field were included. The Deputy Director-General of the China CDC, Dr. George F. Gao, worked in Sierra Leone as the co-team leader of the first team of the mobile laboratory for 2 months (Gao and Feng, 2014). He represented an idol to recruit more young health experts to work on the ground in Africa (Fig. 2), and I was one of them.

**Figure 2:**
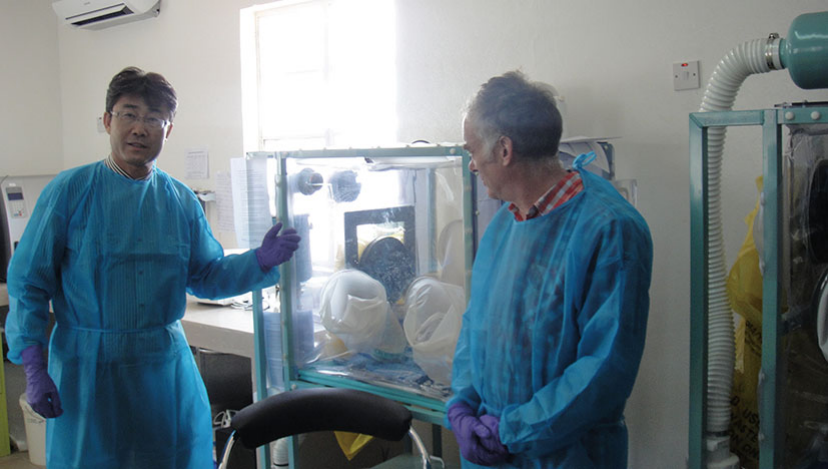
The Deputy Director-General of the China CDC, Dr. George F. Gao, worked in Sierra Leone as the co-team leader of the first team of the mobile laboratory from September to November, 2014

Aside from the mobile laboratory, taking into consideration a long-term goal to support disease control in Western Africa, the Chinese government has built the first fixed P3 lab in West Africa (Jui, Freetown, Sierra Leone). In the middle of April, 2015, I came back to Africa with the second team for the fixed P3 lab, working as the main operator in the core area for two and a half months. Every time that I opened specimen packages in the hood, I reminded myself that the tube in my hand might contain the Ebola virus at a sky-high titer. Indeed, all of my colleagues recognized that biosafety is the priority of the laboratory testing for Ebola. During this period, Ebola virus genomes were quickly assembled and analyzed by the Chinese deep sequencing platform in Western Africa (Tong et al., 2015). This platform in the field makes it possible to trace the origin and transmission chain of the Ebola viruses soon after any new case is reported (Fig. 3).

**Figure 3:**
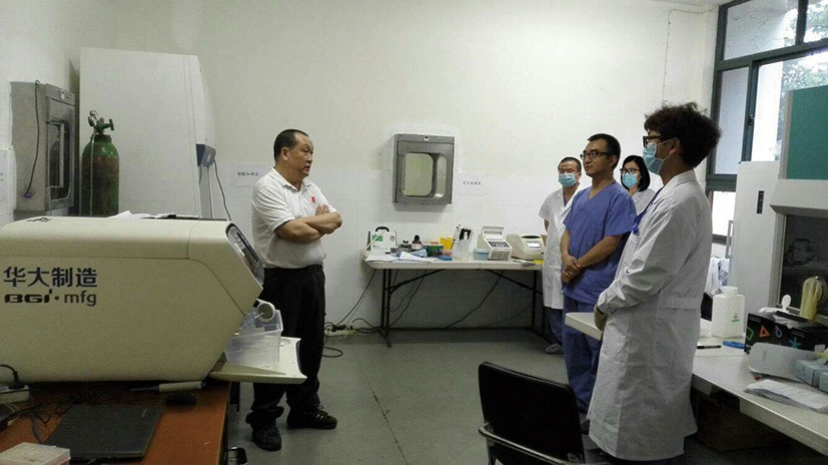
Ebola virus genomes were assembled and analyzed by the Chinese deep sequencing platform in Freetown. The lead of the second team, Professor Yuelong Shu was discussing the sequencing project with the colleagues

With the elapse of new Ebola cases, the Ebola diagnostic laboratories run on international aid were closed one by one. In contrast, China CDC strengthened the laboratory work force to avoid a potential flare-up of the Ebola epidemic. In the end of 2015, I came back to Freetown as the lead of the fourth team for the fixed P3 lab to perform Ebola testing together with nine other Chinese colleagues.

The Ebola surveillance strategy in Sierra Leone was a community-based swab test of dead individuals, together with hospital-based blood testing of suspected cases. As the number of specimens to be tested increased, the human resources in the laboratory became insufficient. However, our team accomplished its Ebola testing job and established good coordination with Dr. Abdul Kamara, who is the National Laboratory Services Manager of the Ministry of Health and Sanitation (MOHS), Sierra Leone (Fig. 4).

**Figure 4:**
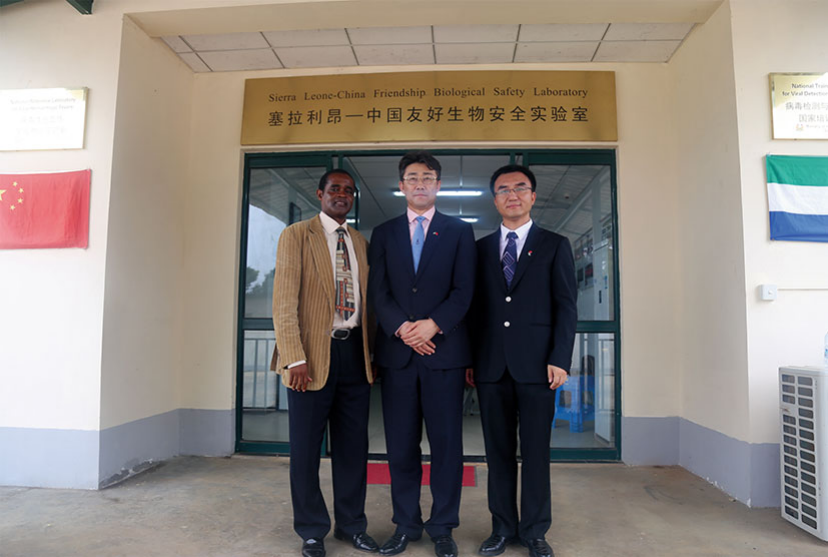
Photograph of Dr. George F. Gao (middle) with Dr. Abdul Kamara (left) and myself (right) during Dr. Gao’s second journey to Sierra Leone

In the end of January, 2016, we have helped to diagnose the latest and hopefully the last Ebola case in Sierra Leone. In the meantime, aside from the swab tests of the dead for routine Ebola surveillance, the laboratory have taken part in a project focused on virus persistence in the survivors (Virus Persistence Study, VPS), collaborating with MOHS of Sierra Leone, the WHO and U.S. Centers for Disease Control and Prevention (US-CDC) (Deen et al., 2015). During the post Ebola era, the proper management and care of the Ebola survivors is one of the most important tasks for the control of Ebola. The VPS yielded a good scientific reference for survivor counseling, and this project is also a representative example for international cooperation for disease control in Africa.

In the post-Ebola era, the international support of public health to Western Africa should not be diminished but strengthened. The risk of a flare-up of Ebola still exists (Wong et al., 2016), considering the unknown reservoir of Ebola viruses that may persist in animal hosts. Furthermore, the disease surveillance capacity of many African countries is yet unbelievable  weak, and the risk of importing yellow fever and Zika virus also exists. Thus, China CDC quickly reacted to this global health situation and empowered the fixed P3 lab with the capacity to test for yellow fever virus and Zika virus in West Africa in March, 2016.

In addition to the direct support for disease control, the fixed P3 lab is responsible for training local specialists for disease control, which is the foundation of long-term support for Africa. In the past year, several Sierra Leonian specialists have received training in the laboratory, including theory and practice in the fixed P3 lab and field training in China. They now work as the team members together with Chinese colleagues, which facilitated the establishment of a long-term co-work strategy in the laboratory. When I was invited to the wedding of one of my Sierra Leonian colleagues, Mr. Gerald Bagura, he mentioned that the team in the laboratory worked like a family (Fig. 5), which was the highest praise for the teamwork in the fixed P3 lab.

**Figure 5:**
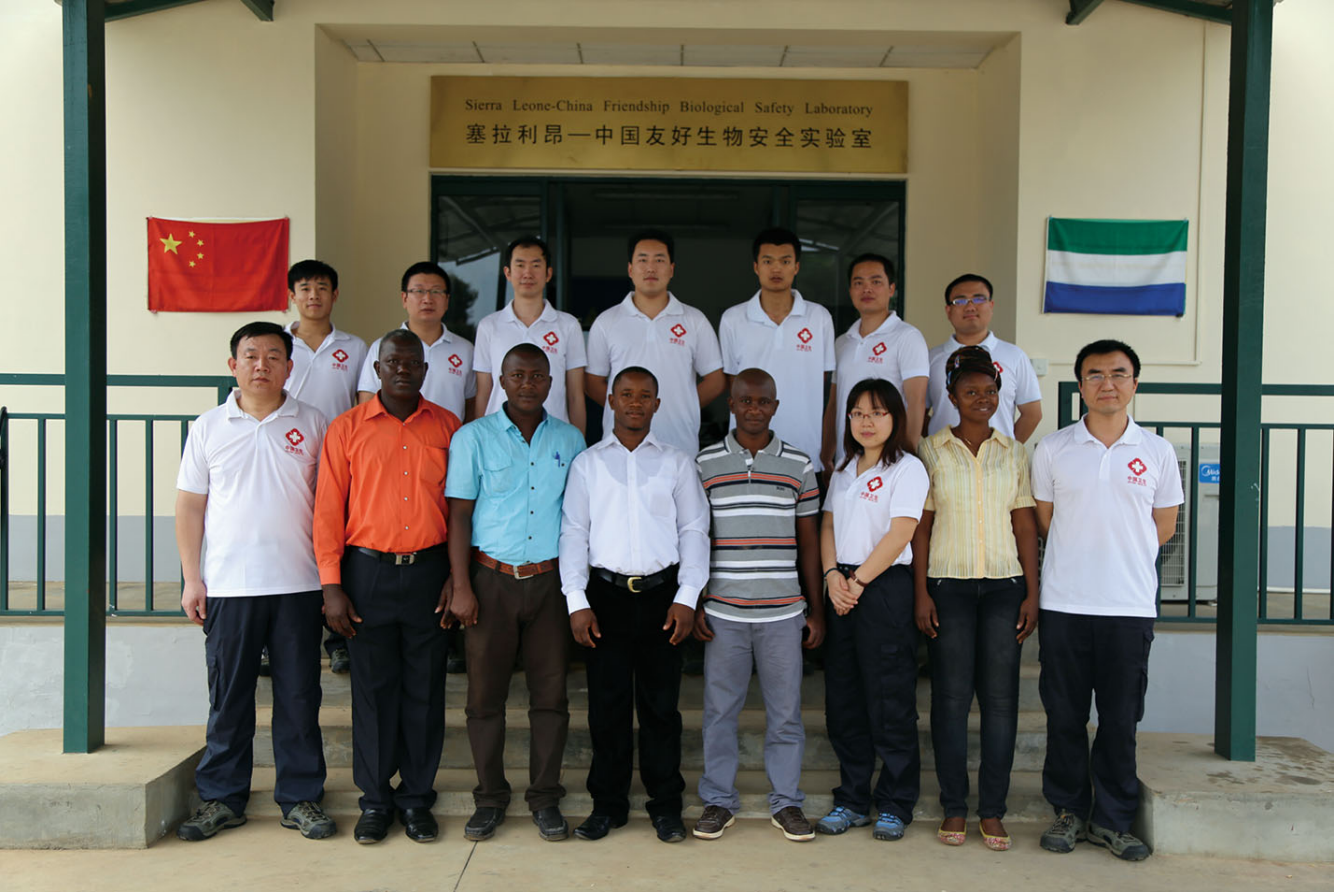
The fourth Ebola test team from China working in the fixed P3 lab together with their Sierra Leonian colleagues

From its commencement in February 2015, the fixed P3 lab has been playing an important role in virus detection and training the local health workforce. Based on the previous contribution and the current capacity of the laboratory, the MOHS of Sierra Leone authorized the designation of the laboratory as the “National Reference Laboratory for Viral Hemorrhagic Fevers” and the “National Training Center for Virus Detection and Biosafety” in the end of June, 2016, before our team left the country. Dr. Gao was invited back to Freetown to attend the unveiling ceremony (Fig. 4). He gave a speech to emphasize the developing orientation of the P3 lab. Dr. Gao also talked with the president of Sierra Leone, Ernest Bai Koroma, about the possibility for China CDC to strengthen our support of the research on and pre-warning of tropical diseases.

Supporting the people of Western Africa during the Ebola epidemic is the first step of the action ‘moving the disease control frontline onto the “battlefield” anywhere in the world’ of the China CDC. In the future, additional young scientists and specialists in disease control are needed to work on the ground of Africa. I am proud to have witnessed this process and contributed a little based on what I learned over the past few years. The trip from public health to global health has just begun.

## References

[CR1] Baize S, Pannetier D, Oestereich L, Rieger T, Koivogui L, Magassouba N, Soropogui B, Sow MS, Keita S, De Clerck H (2014). Emergence of Zaire Ebola virus disease in Guinea. N Engl J Med.

[CR2] Deen GF, Knust B, Broutet N, Sesay FR, Formenty P, Ross C, Thorson AE, Massaquoi TA, Marrinan JE, Ervin E et al (2015) Ebola RNA persistence in semen of ebola virus disease survivors—preliminary report. N Engl J Med. doi:10.1056/NEJMoa1511410

[CR3] Gao GF, Feng Y (2014). On the ground in Sierra Leone. Science.

[CR4] Tong YG, Shi WF, Liu D, Qian J, Liang L, Bo XC, Liu J, Ren HG, Fan H, Ni M (2015). Genetic diversity and evolutionary dynamics of Ebola virus in Sierra Leone. Nature.

[CR5] Wong G, Gao GF, Qiu X (2016). Can Ebola virus become endemic in the human population?. Protein Cell.

